# Drugs utilization profile in England and Wales in the past 15 years: a secular trend analysis

**DOI:** 10.1186/s12875-022-01853-1

**Published:** 2022-09-16

**Authors:** Abdallah Y. Naser, Hassan Alwafi, Tamara Al-Daghastani, Sara Ibrahim Hemmo, Hamzeh Mohammad Alrawashdeh, Zahraa Jalal, Vibhu Paudyal, Nawras Alyamani, Murouj Almaghrabi, Ahmad Shamieh

**Affiliations:** 1 Department of Applied Pharmaceutical Sciences and Clinical Pharmacy, Faculty of Pharmacy, Isra University, Amman, Jordan; 2 Faculty of Medicine, Umm Al-Qura University, Mecca, Saudi Arabia; 3 Department of Medical Allied Sciences, Al-Balqa Applied University, Al-Salt, Jordan; 4 Department of Ophthalmology, Sharif Eye Centers, Irbid, Jordan; 5 School of Pharmacy, Institute of Clinical Sciences, University of Birmingham, Birmingham, UK; 6 Daniel Castro Dental Clinics, El Paso, TX USA

**Keywords:** Drugs utilization, England, Medications, United Kingdom, Wales

## Abstract

**Background:**

Medication use assessment has a critical role in promoting the effective and rational use of pharmaceutical medications. There are no studies that have explored the utilization of all medications in England and Wales in the past 15 years without restrictions in the age group being studied or class of medications.

**Aim:**

To explore the medication utilization pattern of dispensed medications in England and Wales in the past 15 years.

**Method:**

A secular trend analysis study using publically available dispensing data on the population level in England and Wales for the duration between 2004 and 2019. Medication dispensing data was extracted from the Prescription Cost Analysis database.

**Results:**

Medication prescriptions rate increased by 42.6% [from 1,345,095.75 (95% CI 1,345,004.25 – 1,345,187.26) in 2004 to 1,918,138.48 (95% CI 1,918,038.38 – 1,918,238.57) in 2019 per 100,000 persons, trend test, *p* < 0.001]. During the study period, the most common medication prescriptions were for the cardiovascular system, central nervous system, and endocrine system, which accounted for 30.2%, 18.8%, and 9.4%, respectively. The rate of medication prescriptions for skin, immunological products and vaccines, infections, and musculoskeletal and joint diseases decreased by 18.4%, 15.8%, 9.8%, and 5.7%, respectively.

**Conclusion:**

The last two decades have witnessed a remarkable rise in the quantity of medications dispensed in community settings. Utilization of chronic disease medications has increased in the past 15 years, specifically, dispensed medications for the cardiovascular system, central nervous system, and endocrine system. It is necessary to conduct additional cohort studies to investigate the clinical outcomes and prescribing safety of these medications.

**Supplementary Information:**

The online version contains supplementary material available at 10.1186/s12875-022-01853-1.

## Background

The estimated global expenditure on prescription medications in 2020 is $1.3 trillion [1]. It is anticipated that these high spending rates will rise by 3–6% annually across the world [2]. In the United Kingdom (UK), the National Health Service (NHS) significantly relies on primary care physicians to evaluate patients with a variety of presentations [3]. A total of £8.8 billion, or 8.3% of the NHS's annual budget, was spent in 2011 on the over 1 billion prescriptions that general practitioners dispensed [4]. The cost of community prescription medication dispensing in England in 2020 was £9.61 billion, with a total of 1.11 billion prescription items dispensed. Since effective medical care is impossible without the necessary medications, medications play a vital role in healthcare and are an essential component of it [5, 6]. Medication therapy prevents epidemics and diseases in addition to improving health and saving lives [6]. Around half of patients fail to take their prescribed medications as directed, and more than half of all medications are sold, distributed, or prescribed improperly worldwide [7]. The risk of medication errors and adverse reactions increases with irrational medication use, which includes the use of multiple medications (polypharmacy), improper antimicrobial agent use, excessive use of injectable dosage forms, and noncompliance with clinical guidelines recommendations [8–10]. Therefore, to receive the maximum benefit, medications must be used rationally [11].

In the UK, between 1999 and 2009, the yearly number of prescriptions dispensed increased by 65%, from about 653 million to 1,074 million (from 11 per person to 17.4 per person) [12, 13]. Medication use assessment has a critical role in promoting the effective and rational use of pharmaceutical medications [14, 15]. Determining medication consumption and prescription patterns affords beneficial feedback to prescribers to advance their prescribing practice [16]. Prescription analyzing studies are essential for clinicians and policymakers as they help them determine preferences for improving rational medication use at the national level [16, 17]. Previous research in the UK investigated the prescribing practices for particular classes of medications, including antidiabetic medications, antipsychotics, acid suppressants, and medications for cardiovascular diseases [18–22], but there is no previous study that has explored the prescribing pattern for all medications. Therefore, this study aimed to explore the trend of medications prescribing in England and Wales in the past 15 years.

## Methods

### Study design

This was a secular trend analysis study on the population level using prescribing data in England and Wales for the period between 2004 and 2019.

### Data source

Medication prescription data were extracted from the Prescription Cost Analysis (PCA) database National Health Services (NHS) Business Services Authority [23]. The PCA database contains data on all medications that are prescribed by general practitioners (GP)s and other healthcare specialists, including hospital doctors, pharmacists, and nurses, and are then given out to the public in England and Wales by dispensing doctors, pharmacy contractors, or appliance contractors [23, 24]. Patients’ demographic information is not included in this database, which only gives aggregate data on the total number of prescriptions dispensed.

This database reports data regarding medication prescribing based on the British National Formulary (BNF) therapeutic classification system [25]. The BNF is a reference book for healthcare professionals in the UK that provides a wide range of information and suggestions on prescribing and pharmacology, as well as specific information about various medications that are available through the UK NHS. The BNF contains information on indications, contraindications, adverse effects, dosages, legal categorization, names, and costs of readily available proprietary and generic formulations, as well as any other noteworthy details. It serves as a reference for the proper dosage, indication, interactions, and side effects of medications and is used by pharmacists, doctors (both GPs and specialist practitioners), and other prescribing healthcare professionals (such as nurses, pharmacy technicians, paramedics, and dentists). It is divided into several categories, the majority of which are organized by body system and cover medications and preparations [26]. The NHS Prescription Services employs a therapeutic classification system that closely adheres to and is based on the BNF, but there are times when this is not feasible. All items that may be prescribed for use on the NHS in England must be covered by the chapter, paragraph headings, and classification of medications and appliances used by the NHS Prescription Services and the Prescribing Toolkit. As a result, they might not always be an exact equivalent of the BNF. There may be minor changes to the BNF chapter and section headings. The NHS Prescription Services has created additional Pseudo BNF chapters, sections, paragraphs, etc. to either reclassify items or to accommodate preparations not listed in the BNF, such as unlicensed medicines. Items detailed in the BNF may be classified by the NHS Prescription Services under pseudo BNF classifications [27].

Prescriptions data covers all medications that were dispensed in the community in England and Wales [23]. The BNF classification system (chapters one through fifteen) was used to identify medication prescribing data. Prescriptions reported in the PCA include those written by pharmacists, doctors, dentists, and nurses. Some medications for minor diseases can be purchased in the UK without a prescription (over-the-counter). Contrarily, prescription-only drugs need to be prescribed by a qualified healthcare professional. This individual could be a GP, hospital doctor, pharmacist, dentist, nurse, optometrist, physiotherapist, or podiatrist [28].

### Data analysis

Annual rates of medication prescriptions with their 95% confidence intervals (CIs) were calculated using the number of prescriptions for each medication divided by the total mid-year population. The trends for medication prescriptions were assessed using a Poisson model. A two-sided *p* < 0.05 was considered statistically significant. All analyses were performed using Statistical Package for Social Science software version 27 (IBM Corp, Armonk, NY, USA).

## Results

**Fig. 1:**
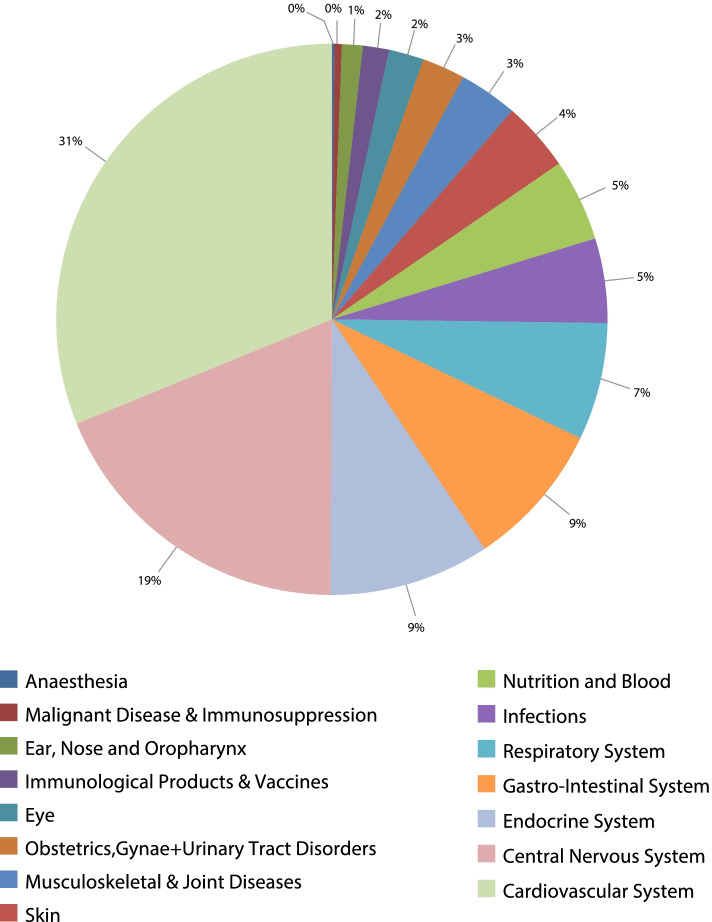
Percentage of each medication prescription from the total number of medication prescriptions

The absolute number of prescription items dispensed annually in England and Wales for all causes increased by 58.7% from 718,499,038 in 2004 to 1,140,138,443 in 2019, representing an increase in medication prescription rate of 42.6% [from 1,345,095.75 (95% CI 1,345,004.25 – 1,345,187.26) in 2004 to 1,918,138.48 (95% CI 1,918,038.38 – 1,918,238.57) in 2019 per 100,000 persons, trend test, *p* < 0.001]. The most common medication prescriptions were for the cardiovascular system, central nervous system, and endocrine system, which accounted for 30.2%, 18.8%, and 9.4%, respectively (Table 1). Figure 1 presents percentage of each medication prescription from the total number of medication prescriptions.

**Table 1:** The most commonly dispensed medication in the same therapeutic class

BNF chapter code	Therapeutic class	The most commonly dispensed medication in the same therapeutic class	Percentage from total number of prescriptions in the same therapeutic class
01	Gastro-intestinal system medications	Antisecretory drugs and mucosal protectants	60.3%
02	Cardiovascular system medications	Hypertension and heart failure medications	21.8%
03	Respiratory system medications	Bronchodilators medications	45.3%
04	Central nervous system medications	Analgesics	35.4%
05	Infections medications	Antibacterial drugs	85.8%
06	Endocrine system medications	Drugs used in diabetes	46.5%
07	Obstetrics, gynaecology, and urinary-tract disorders medications	Drugs for genito-urinary disorders	54.3%
08	Malignant disease and immunosuppression medications	Sex hormones and hormone antagonists in malignant disease	61.8%
09	Nutrition and blood-related products	Vitamins	44.7%
10	Musculoskeletal and joint diseases medications	Drugs used in rheumatic diseases and gout	76.0%
11	Eye medications	Treatment of glaucoma	41.8%
12	Ear, nose, and oropharynx medications	Drugs acting on the nose	63.2%
13	Skin medications	Emollient and barrier preparations	37.0%
14	Immunological products and vaccines	Vaccines and antisera	100.0%
15	Anaesthesia	Local anaesthesia medications	86.8%

During the past two decades, a large increase in the rate of medication prescriptions was noticed in nutrition and blood with 1.53-fold. Furthermore, the rate of medication prescriptions for anaesthesia, endocrine system, gastro-intestinal system, and obstetrics, gynaecology, and urinary-tract disorders increased by 87.1%, 83.7%, 74.6%, and 70.2%, respectively. However, the rate of medication prescriptions for skin, immunological products and vaccines, infections, and musculoskeletal and joint diseases decreased by 18.4%, 15.8%, 9.8%, and 5.7%, respectively (Table 2). Figure 1S presents prescription rates of all medications in England and Wales between 2004 and 2019.

**Table 2:** Change in the prescription rate of medications by therapeutic class

Therapeutic class	Rate of prescribing in 2004 per 100,000 persons (95% CI)	Rate of prescribing in 2019 per 100,000 persons (95% CI)	Percentage change from 2004 – 2019
Gastro-intestinal system medications	103,465.69 (103,439.86 – 103,491.52)	180,617.77 (180,586.84 – 180,648.69)	74.6%
Cardiovascular system medications	407,305.61 (407,263.94 – 407,347.28)	584,695.14 (584,655.52 – 584,734.76)	43.6%
Respiratory system medications	102,509.91 (102,484.19 – 102,535.64)	130,550.51 (130,523.43 – 130,577.60)	27.4%
Central nervous system medications	247,629.78 (247,593.18 – 247,666.39)	381,689.81 (381,650.75 – 381,728.86)	54.1%
Infections medications	82,616.61 (82,606.44 – 82,626.77)	74,498.74 (74,487.66 – 74,509.82)	-9.8%
Endocrine system medications	109,310.42 (109,283.96 – 109,336.88)	200,804.09 (200,771.88 – 200,836.29)	83.7%
Obstetrics, gynaecology, and urinary-tract disorders medications	32,262.91 (32,250.38 – 32,275.45)	54,914.09 (54,901.44 – 54,926.74)	70.2%
Malignant disease and immunosuppression medications	8,029.08 (8,021.80 – 8,036.37)	8,385.11 (8,378.07 – 8,392.16)	4.4%
Nutrition and blood-related products	42,954.03 (42,936.83 – 42,971.22)	108,515.77 (108,490.76 – 108,540.77)	152.6%
Musculoskeletal and joint diseases medications	59,759.91 (59,746.76 – 59,773.06)	56,369.73 (56,357.12 – 56,382.34)	-5.7%
Eye medications	31,691.22 (31,678.75 – 31,703.70)	33,348.64 (33,336.65 – 33,360.62)	5.2%
Ear, nose, and oropharynx medications	18,844.39 (18,833.90 – 18,854.88)	20,726.21 (20,715.90 – 20,736.51)	10.0%
Skin medications	69,938.78 (69,926.49 – 69,951.08)	57,095.46 (57,082.88 – 57,108.05)	-18.4%
Immunological products and vaccines	27,124.69 (27,112.77 – 27,136.62)	22,835.83 (22,825.15 – 22,846.50)	-15.8%
Anaesthesia	1,652.72 (1,649.30 – 1,656.14)	3,091.59 (3,087.19 – 3,095.99)	87.1%

Table 1 below highlights the most commonly dispensed medication class for each therapeutic class as per the BNF therapeutic classification system.

### Prescription rate of medications by therapeutic class

**Fig. 2:**
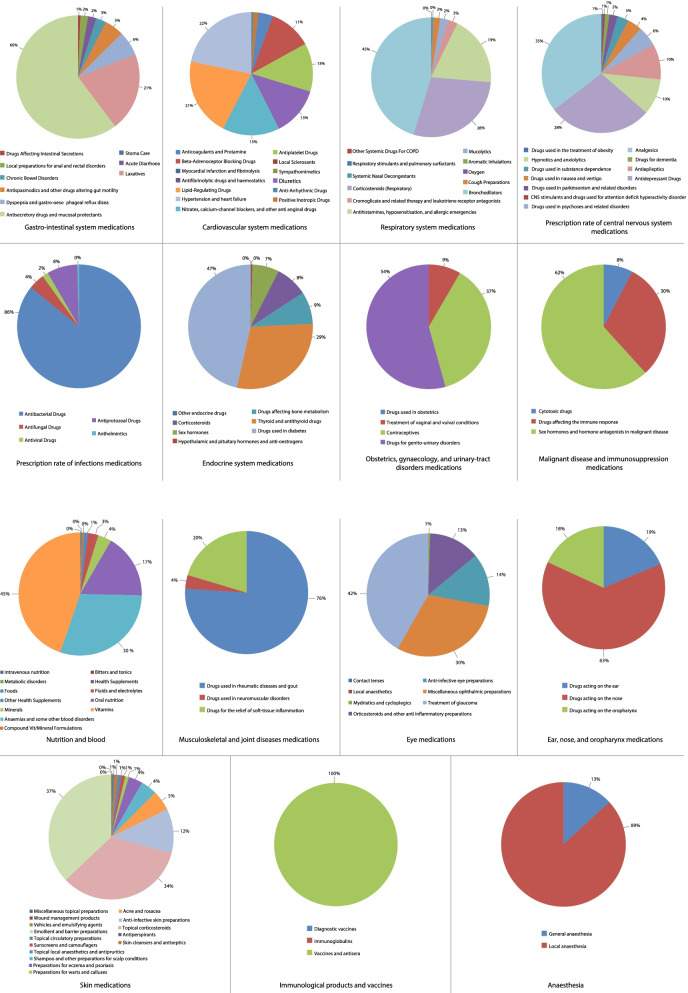
Percentage of each medication prescription from the total number of medications in the same therapeutic class between 2004 and 2019

**Fig. 3:**
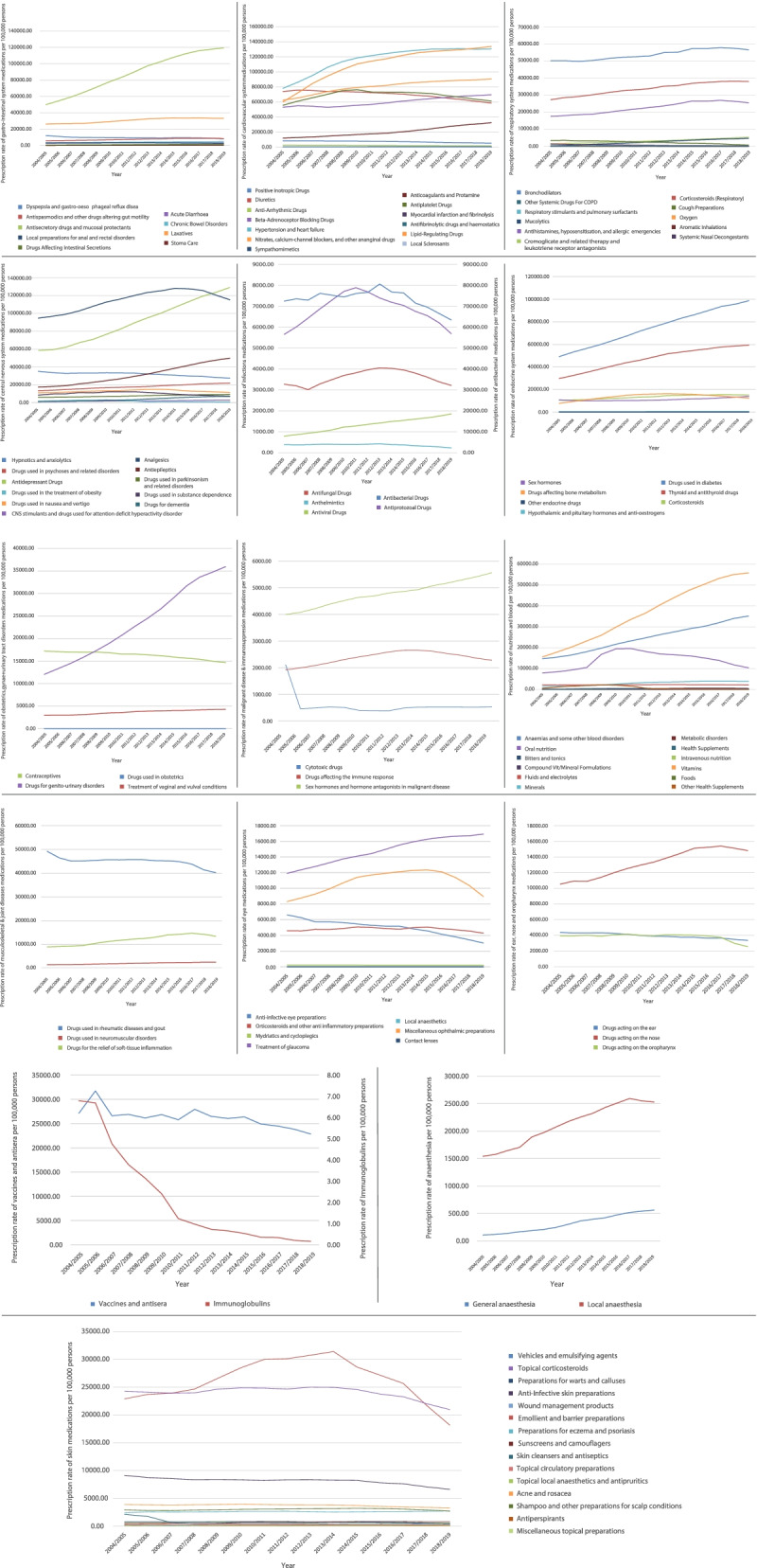
Prescription rate for each medication stratified by therapeutic class

Figure 2 presents the percentage of each medication prescription from the total number of medications in the same therapeutic class between 2004 and 2019. Table 2 below presents the change in the prescribing rate for all therapeutic classes during the past 15 years in England and Wales. Regarding the medications related to the gastro-intestinal system, the overall non-adjusted prescribing rate of medications increased by 74.6%. The overall prescribing rate of cardiovascular system medications increased by 43.6%, Fig. 3. The overall prescribing rate of central nervous system medications increased by 54.1%. The overall prescribing rate of endocrine system medications increased by 83.7% during the study period. The overall prescribing rate of obstetrics, gynaecology, and urinary-tract disorder medications increased by 70.2%. For further details on the change of prescription rate for other therapeutic classes please refer to the supplementary file, Table S1.

## Discussion

An increase of 42.6% in the rate of medication prescriptions was noted among all medication therapeutic classes from 2004 to 2019. The most common medication prescriptions were for the cardiovascular system, followed by the central nervous system, and the endocrine system. Among all the dispensed medications within the systems, we found that anti-secretory medications and mucosal protectants (60.3%), genito-urinary disorders medications (54.3%), and antidiabetic medications (46.5%) were noted to be the most commonly dispensed. According to several studies that examined the overall trends of medication prescription, the frequency of prescribing medications for various age groups has increased globally over the past two decades [29–32]. Previous studies in Denmark, Sweden, and Norway explored the utilisation pattern of different classes of medications such as opioids and antiarrhythmic medications and reported an increase in their utilisation [33–35]. Recent studies in the UK showed an increase in hospitalizations for various acute and chronic conditions, such as viral diseases, respiratory diseases, and heart diseases [19, 36, 37]. Raised hospitalisation rates highlight that there is an increase in the prevalence of these diseases that lead to hospitalisation, which itself could lead to an increase in the prescribing pattern of medications.

Our study revealed that prescriptions for the cardiovascular system were the most common across all systems (30.2%). In particular, hypertension and heart failure medications were the most commonly dispensed. This is in line with the rising prevalence of cardiovascular diseases (CVDs), which nearly doubled from 271 million in 1990 to 523 million in 2019 [38]. In addition, there are an estimated 1.28 billion adults worldwide who have hypertension and 64,34 million cases of heart failure (8.52 per 1,000 persons) [39]. In 2017, in England, it is estimated that around 11.8 million adults had hypertension, which is equal to 26.2% of the adult population [40], with an overall estimate in the UK of 33.0% [41]. Between males and females, there are disparities in the use of cardiovascular medications [42]. This could be due to several reasons including the fact that females are more health-conscious and frequently prescribed medications [43, 44]. On the other hand, a recent large scale meta-analysis explored sex differences in cardiovascular medication prescriptions in primary care settings of more than 2 million patients and reported that prescription rate for aspirin, statin, and antihypertensive medications was higher among males compared to females [45].

A previous study by Hemmo et al. has explored the hospitalisation pattern for ischemic heart diseases (IHD)s and the prescription pattern for CVD medication for the period between 1999 and 2019 [19]. Their study found that the prescription rate for beta-blockers, calcium channel blockers, anti-platelets, and lipid-lowering medications, was negatively correlated with IHD-related hospital admission rates [19]. The prescription rate for these medications increased as they are mainly maintenance therapy and have been shown to improve symptoms and prolong survival [46–50]. The prescription rate for CVD medications in our study increased for several reasons, which included advancements in the development of medications and the diagnosis of the disease, as well as modifications to practice guidelines [19, 51]. Besides, the diagnostic approach for coronary artery disease has been fundamentally altered with the emergence of coronary computed tomography angiography (CCTA) and coronary artery calcium score (CACS), and their widespread usage as anatomical diagnostic tools since 2011. Additionally, CVD has been diagnosed earlier with an anatomical method than with the traditional functional technique. CACS has the ability to identify non-obstructive plaques on CCTA even before symptoms appear [52].

We found that medications dispensed for CNS conditions were the second most commonly dispensed (18.8%) after analgesics (28.1%). Only 0.6% of the study population has been prescribed CNS stimulant medications, which indicates good practice and caution when describing such medications. The high use of antidepressants is not a new finding, as a previous study in the US has reported that antidepressants are among the most frequently used medications [53]. A previous study in the UK reported that 57.8% of elderly patients with multiple comorbidities who take more than five medications (polypharmacy) were prescribed CNS medications [54]. Patients with polypharmacy were more likely to be prescribed opioid and non-opioid analgesics, tricyclic antidepressants, and selective serotonin re-uptake inhibitors [54]. Aging is another factor that is associated with the increase in the prevalence of CNS disorders and the utilization of their associated medications [55, 56]. The proportion of people aged 65 and over in the UK is expected to rise from 18.5% in 2019 to 23.9% by 2039. With such a rise in the elderly population, the socioeconomic burden of age-related health conditions will rise, necessitating effective preventive or therapeutic interventions [57–61]

Regarding the third most dispensed medications, 9.4% of prescriptions were for endocrine system disorders. Our results showed that the rate of endocrine medication prescriptions has increased significantly in the past two decades. The most commonly dispensed medications were antidiabetic medications (46.5%). The World Health Organization (WHO) statistics showed that the number of newly diagnosed cases of diabetes increased from 108 million in 1980 to 422 million in 2014 [62]. In the UK, it is estimated that 6% of the population has diabetes (diagnosed and undiagnosed) [63]. In addition, a previous study conducted by Saeedi et al*.* [64] reported that the total number of patients with diabetes worldwide was 463 million in 2019, representing 9.3% of the global adult population (20–79 years). By 2035, this number is expected to rise to 592 million [65]. These facts indicate that the number of diabetes mellitus cases will increase steadily with time, which indicates the need for healthy lifestyle awareness. Furthermore, healthcare providers must be aware of non-pharmacological treatment options such as diet and exercise [66, 67].

Antibiotic overuse is one of the contributing factors to developing antibiotic resistance, including the creation of multidrug-resistant bacteria (superbugs), leading to life-threatening infections [68]. Antibiotic resistance is one of the top ten public health concerns confronting humanity [69]. Our study revealed that the most commonly dispensed medications for infections were antibacterial (85.8%), and the highest rate of prescription was determined between 2012–2013. This may be attributed to antibiotic misuse as demonstrated by several studies [70–72]. Using antibiotics as prophylaxis in some conditions or during surgeries can also explain the high prescription rate. In our study, we found a decrease in the dispensing rate of antimicrobial agents of 9.8%. Many initiatives have been introduced to reduce antimicrobial prescribing in the UK. One of them is the Scottish Reduction in Antimicrobial Prescribing (ScRAP) programme [73]. Additionally, the improvement in the National Institute for Health and Care Excellence (NICE) guidelines and the implementation of a nationwide incentive program contributed to the decline in the amount of antibiotics prescribed for respiratory infections [74, 75].

To the best of our knowledge, this is the first study of its kind to examine the patterns of medications use across all therapeutic classes in England, as opposed to earlier studies that focused on a particular class of medications. This will improve the usefulness of our research findings and provide us with a better understanding of the current medications utilisation status. This will make it easier to identify therapeutic areas with high dispensing rates for planning and development of efforts aimed at improving them. The current study has several limitations. This is a secular trend study using publicly available data provided by PCA databases on the population level, not on the individual level. Therefore, we were not able to identify patients’ medical history and other important confounders (such as age, gender, and comorbidities) that might influence medications utilisation pattern. The used database records the number of prescriptions dispensed for multiple times, therefore, this might contribute to an overestimation. We did not have data on the gender or age of patients who dispensed medications in our study. This restricted our ability to explore gender and age-based medication utilization patterns. Therefore, our findings should be interpreted carefully.

We recommend future studies to investigate medications utilization patterns in other countries. Future studies should aim at exploring improper use of medications. These efforts should be directed towards different therapeutic classes, including gastro-intestinal system medications, cardiovascular system medications, central nervous system medications, obstetrics, gynaecology, and urinary-tract disorders medications, and endocrine system medications, which showed the highest increase in the rate of dispensing in our study. Further studies in this area are of high importance, especially those who are exploring the number of medications used on an individual level (to detect cases of polypharmacy). This is important for a better understanding of the issue, which will help in suggesting suitable cost-effective solutions.

## Conclusion

The last two decades have witnessed a remarkable rise in the prescribing of medications. Utilization of chronic disease medications has increased in the past 15 years, specifically, dispensed medications for the cardiovascular system, central nervous system, and endocrine system. Our results showed an increase in the frequencies and rates of medications dispensed among different medical specialties (different therapeutic classes), which might provide a good reference for further studies to raise awareness towards medication practices. It is necessary to conduct additional cohort studies to investigate the clinical outcomes and prescribing safety of these medications. This will ultimately enhance the utilization of medications.   

## Supplementary Information

**Additional file 1:** **Table S1.** Change in the prescription rate of medications bytherapeutic class. **Figure 1S.** Prescription rates of all medications inEngland and Wales between 2004 and 2019.

## Data Availability

Publicly available datasets were analyzed in this study. This data can be found here: https://www.nhsbsa.nhs.uk/prescription-data/dispensing-data/prescription-cost-analysis-pca-data.

## Abbreviations

BNF: British National Formulary
GP: General Practitioner
PCA: Prescription Cost Analysis
UK: United Kingdom
SPSS: Statistical package for social sciences
IHD: Ischemic Heart Disease
NHS: National Health Services
WHO: World Health Organization
CNS: Central Nervous System
CI: Confidence interval
CVD: Cardiovascular Diseases
CCTA: Coronary Computed Tomography Angiography
CACS: Coronary Artery Calcium Score
NICE: National Institute for Health and Care Excellence
ScRAP: Scottish Reduction in Antimicrobial Prescribing
