# Supplementary material for: Drugs utilization profile in England and Wales in the past 15 years: a secular trend analysis
Source: BMC Prim Care. 2022 Sep 16;23:239. doi: 10.1186/s12875-022-01853-1 (PMC9482186; doi:10.1186/s12875-022-01853-1)
Supplement: Supplementary file 1 — Additional file 1: Table S1. Change in the prescription rate of medications bytherapeutic class. Figure 1S. Prescription rates of all medications inEngland and Wales between 2004 and 2019. [file 12875_2022_1853_MOESM1_ESM.docx]

**Supplementary file:**

**Details on the change of prescription rate for other therapeutic classes**

Regarding the medications related to the gastro-intestinal system, the overall non-adjusted prescribing rate of medications increased by 74.6%, stoma care related medications prescribing rate increased by 2.25-fold, followed by drugs affecting intestinal secretions and antisecretory drugs and mucosal protectants which increased by 1.84-fold and 1.39-fold, respectively. The overall prescribing rate of cardiovascular system medications increased by 43.6%. The highest increase in the prescribing rate for cardiovascular system medications was noticed for sympathomimetics, followed by anticoagulants and protamine, and lipid-regulating medications, with an increase of 20.88-fold, 1.70-fold, and 1.22-fold, respectively, Figure 4. The overall prescribing rate of central nervous system medications increased by 54.1%. The highest increase in the prescribing rate for these medications was noticed for medications for dementia, central nervous system (CNS) stimulants and medications used for attention deficit hyperactivity disorder, and antiepileptics with 6.1-fold, 2.05-fold, and 1.87-fold, respectively. The overall prescribing rate of endocrine system medications increased by 83.7% during the study period. The highest increase in the prescribing rate for these medications was noticed for medications used for diabetes, thyroid and antithyroid medications, and medications affecting bone metabolism, which accounted for 1.01-fold, 1.00-fold, and 60.4%, respectively.

The overall prescribing rate of obstetrics, gynaecology, and urinary-tract disorder medications increased by 70.2%. The highest increase in the prescribing rate for these medications was noticed for drugs for genito-urinary disorders and treatment of vaginal and vulval conditions, with 1.98-fold and 43.8%, respectively. The overall prescribing rate of malignant disease and immunosuppression medications increased by 4.4%. The prescription rate for sex hormones and hormone antagonists in malignant disease, as well as drugs affecting immune response, increased by 39.2% and 18.9%, respectively. Sex hormones and hormone antagonists in malignant disease and drugs affecting the immune response prescriptions rate increased by 39.2% and 18.9%, respectively.

The overall prescribing rate of respiratory system medications increased by 27.4%. Mucolytics, cromoglicate and related therapy and leukotriene receptor antagonists, and antihistamines, hyposensitisation, and allergic emergencies showed the highest increase in the prescriptions rate in this category with 21.83-fold, 2.84-fold, and 45.8%, respectively.

The overall prescribing rate of infections medications decreased by 9.8%. However, the prescriptions rate for antiviral medications increased by 1.34-fold during the study period. The overall prescribing rate of malignant disease and immunosuppression medications increased by 4.4%. Sex hormones and hormone antagonists in malignant disease and drugs affecting the immune response prescriptions rate increased by 39.2% and 18.9%, respectively.

The overall prescribing rate of nutrition and blood-related products increased by 1.53-fold. Compound Vitamin/Mineral Formulations and vitamins prescriptions rate increased by 3.71-fold and 2.58-fold, respectively. The overall prescribing rate of musculoskeletal and joint diseases medications decreased by 5.7%. However, the prescriptions rate for drugs used in neuromuscular disorders and drugs for the relief of soft-tissue inflammation increased by 70.1% and 49.7%, respectively during the study period. The overall prescribing rate of ear, nose, and oropharynx medications increased by 5.2%. The prescriptions rate for drugs acting on the nose increased by 40.7%, while it decreased for drugs acting on the ear and drugs acting on the oropharynx by 23.2% and 35.2%, respectively during the same period. The overall prescribing rate of eye medications increased by 10.0%. The highest increase in the prescribing rate for these medications was noticed for treatment of glaucoma and miscellaneous ophthalmic preparations with 41.9% and 7.8%, respectively.

The overall prescribing rate of skin medications decreased by 18.4%. However, it increased for vehicles and emulsifying agents, sunscreens and camouflagers, and preparations for eczema and psoriasis by 2.96-fold, 94.0%, and 15.2%, respectively. The overall prescribing rate of immunological products and vaccines decreased by 15.8%. Similarly, the prescriptions rate for its sub-class vaccines and antisera and immunoglobulins decreased by 15.8% and 97.6% during the same period. The overall prescribing rate of anaesthesia increased by 87.1%. The highest increase in the prescribing rate for these medications was noticed for general anaesthesia with 4.12-fold compared to local anaesthesia with 63.9%.

**Table S1: Change in the prescription rate of medications by therapeutic class**

| **Therapeutic class** | **Rate of prescribing in 2004 per 100,000 persons (95% CI)** | **Rate of prescribing in 2019 per 100,000 persons (95% CI)** | **Percentage change from 2004 – 2019** |
| --- | --- | --- | --- |
| **Gastro-intestinal system medications** | **103,465.69 (103,439.86 – 103,491.52)** | **180,617.77 (180,586.84 – 180,648.69)** | **74.6%** |
|  | | | |
| Stoma care | 1.43 (1.33 – 1.53) | 4.65 (4.48 – 4.82) | 225.1% |
| Drugs affecting intestinal secretions | 546.56 (544.58 – 548.54) | 1,553.72 (1,550.57 – 1,556.86) | 184.3% |
| Antisecretory drugs and mucosal protectants | 49,949.98 (49,931.51 – 49,968.46) | 119,308.40 (119,282.34 – 119,334.46) | 138.9% |
| Antispasmodics and other drugs altering gut motility | 5,778.92 (5,772.66 – 5,785.18) | 8,519.56 (8,512.47 – 8,526.66) | 47.4% |
| Chronic bowel disorders | 3,256.90 (3,252.14 – 3,261.66) | 4,467.60 (4,462.35 – 4,472.85) | 37.2% |
| Laxatives | 26,169.62 (26,157.83 – 26,181.41) | 33,047.68 (33,035.73 – 33,059.64) | 26.3% |
| Acute diarrhoea | 2,866.95 (2,862.48 – 2,871.43) | 3,192.29 (3,187.83 – 3,196.76) | 11.3% |
| Local preparations for anal and rectal disorders | 3,013.17 (3,008.59 – 3,017.76) | 2,378.94 (2,375.06 – 2,382.81) | -21.0% |
| Dyspepsia and gastro-oesophageal reflux disea | 11,882.15 (11,873.47 – 11,890.83) | 8,144.92 (8,137.96 – 8,151.87) | -31.5% |
|  | | | |
| **Cardiovascular system medications** | **407,305.61 (407,263.94 – 407,347.28)** | **584,695.14 (584,655.52 – 584,734.76)** | **43.6%** |
|  | | | |
| Sympathomimetics | 7.05 (6.83 – 7.28) | 154.28 (153.28 – 155.28) | 2,087.7% |
| Anticoagulants and protamine | 12,014.50 (12,005.78 – 12,023.22) | 32,440.01 (32,428.11 – 32,451.91) | 170.0% |
| Lipid-regulating drugs | 60,198.97 (60,178.80 – 60,219.14) | 133,661.30 (133,633.95 – 133,688.66) | 122.0% |
| Hypertension and heart failure | 78,178.83 (78,156.07 – 78,201.60) | 130,367.47 (130,340.40 – 130,394.53) | 66.8% |
| Nitrates, calcium-channel blockers, and other antianginal drugs | 62,418.09 (62,405.10 – 62,431.08) | 90,447.49 (90,440.02 – 90,454.97) | 44.9% |
| Beta-adrenoceptor blocking drugs | 53,108.11 (53,094.73 – 53,121.49) | 69,638.45 (69,626.76 – 69,650.14) | 31.1% |
| Antifibrinolytic drugs and haemostatics | 620.18 (618.08 – 622.29) | 752.29 (750.10 – 754.49) | 21.3% |
| Antiplatelet drugs | 55,715.35 (55,702.03 – 55,728.67) | 61,395.44 (61,383.07 – 61,407.82) | 10.2% |
| Diuretics | 74,041.91 (74,030.16 – 74,053.67) | 58,651.02 (58,638.50 – 58,663.54) | -20.8% |
| Anti-arrhythmic drugs | 2,673.46 (2,669.13 – 2,677.78) | 1,755.83 (1,752.49 – 1,759.17) | -34.3% |
| Positive inotropic drugs | 8,328.46 (8,321.05 – 8,335.87) | 5,431.54 (5,425.77 – 5,437.30) | -34.8% |
| Local sclerosants | 0.68 (0.61 – 0.75) | 0.01 (0.00 – 0.02) | -98.0% |
| Myocardial infarction and fibrinolysis | - | - | n/a |
|  | | | |
| **Respiratory system medications** | **102,509.91 (102,484.19 – 102,535.64)** | **130,550.51 (130,523.43 – 130,577.60)** | **27.4%** |
|  | | | |
| Mucolytics | 203.09 (201.88 – 204.30) | 4,636.42 (4,631.07 – 4,641.76) | 2,183.0% |
| Cromoglicate and related therapy and leukotriene receptor antagonists | 1,366.51 (1,363.39 – 1,369.62) | 5,242.95 (5,237.29 – 5,248.62) | 283.7% |
| Antihistamines, hyposensitisation, and allergic emergencies | 17,491.81 (17,481.62 – 17,502.00) | 25,501.60 (25,490.52 – 25,512.69) | 45.8% |
| Corticosteroids (Respiratory) | 27,246.10 (27,234.16 – 27,258.04) | 37,947.06 (37,934.72 – 37,959.40) | 39.3% |
| Bronchodilators | 50,132.91 (50,119.50 – 50,146.31) | 56,483.39 (56,470.79 – 56,495.99) | 12.7% |
| Cough preparations | 3,317.63 (3,312.83 – 3,322.44) | 627.58 (625.57 – 629.59) | -81.1% |
| Aromatic inhalations | 45.32 (44.75 – 45.89) | 6.92 (6.71 – 7.13) | -84.7% |
| Systemic nasal decongestants | 1,240.05 (1,237.08 – 1,243.02) | 104.37 (103.55 – 105.19) | -91.6% |
| Respiratory stimulants and pulmonary surfactants | - | - | n/a |
| Other systemic drugs for COPD | - | 0.21 (0.18 – 0.25) | n/a |
| Oxygen | 1,466.51 (1,463.28 – 1,469.73) | - | n/a |
|  | | | |
| **Central nervous system medications** | **247,629.78 (247,593.18 – 247,666.39)** | **381,689.81 (381,650.75 – 381,728.86)** | **54.1%** |
|  | | | |
| Drugs for dementia | 975.91 (973.28 – 978.55) | 6,924.24 (6,917.79 – 6,930.70) | 609.5% |
| CNS stimulants and drugs used for attention deficit hyperactivity disorder | 909.17 (906.62 – 911.71) | 2,768.19 (2,764.02 – 2,772.36) | 204.5% |
| Antiepileptics | 17,322.66 (17,312.51 – 17,332.81) | 49,791.84 (49,779.13 – 49,804.55) | 187.4% |
| Antidepressant drugs | 58,550.40 (58,530.49 – 58,570.31) | 129,285.19 (129,258.22 – 129,312.16) | 120.8% |
| Drugs used in psychoses and related disorders | 13,346.22 (13,337.10 – 13,355.34) | 21,887.00 (21,876.49 – 21,897.52) | 64.0% |
| Drugs used in parkinsonism and related disorders | 5,792.06 (5,785.79 – 5,798.32) | 9,124.69 (9,117.37 – 9,132.01) | 57.5% |
| Analgesics | 94,789.31 (94,764.47 – 94,814.16) | 115,522.57 (115,496.87 – 115,548.27) | 21.9% |
| Drugs used in nausea and vertigo | 11,020.64 (11,012.24 – 11,029.04) | 11,317.96 (11,309.91 – 11,326.02) | 2.7% |
| Drugs used in substance dependence | 8,351.08 (8,343.66 – 8,358.50) | 7,129.09 (7,122.55 – 7,135.63) | -14.6% |
| Hypnotics and anxiolytics | 35,134.49 (35,121.69 – 35,147.29) | 27,278.54 (27,267.22 – 27,289.86) | -22.4% |
| Drugs used in the treatment of obesity | 1437.84 (1434.64 – 1441.03) | 660.48 (658.43 – 662.54) | -54.1% |
|  | | | |
| **Infections medications** | **82,616.61 (82,606.44 – 82,626.77)** | **74,498.74 (74,487.66 – 74,509.82)** | **-9.8%** |
|  | | | |
| Antiviral drugs | 791.46 (789.09 – 793.84) | 1,853.28 (1,849.85 – 1,856.71) | 134.2% |
| Antiprotozoal drugs | 5,663.64 (5,657.44 – 5,669.84) | 5,694.89 (5,689.00 – 5,700.78) | 0.6% |
| Antifungal drugs | 3,275.20 (3,270.42 – 3,279.97) | 3,220.08 (3,215.59 – 3,224.57) | -1.7% |
| Antibacterial drugs | 72,506.00 (72,494.03 – 72,517.98) | 63,505.06 (63,492.82 – 63,517.30) | -12.4% |
| Anthelmintics | 380.30 (378.65 – 381.95) | 225.42 (224.22 – 226.63) | -40.7% |
|  | | | |
| **Endocrine system medications** | **109,310.42 (109,283.96 – 109,336.88)** | **200,804.09 (200,771.88 – 200,836.29)** | **83.7%** |
|  | | | |
| Drugs used in diabetes | 49,208.37 (49,194.96 – 49,221.78) | 98,672.48 (98,669.57 – 98,675.39) | 100.5% |
| Thyroid and antithyroid drugs | 29,833.34 (29,821.07 – 29,845.61) | 59,504.23 (59,491.75 – 59,516.71) | 99.5% |
| Drugs affecting bone metabolism | 7,829.81 (7,822.61 – 7,837.01) | 12,555.94 (12,547.51 – 12,564.36) | 60.4% |
| Corticosteroids | 10,679.28 (10,671.00 – 10,687.57) | 15,242.75 (15,233.61 – 15,251.89) | 42.7% |
| Sex hormones | 10,944.40 (10,936.03 – 10,952.77) | 13,995.34 (13,986.52 – 14,004.16) | 27.9% |
| Hypothalamic and pituitary hormones and anti-oestrogens | 615.60 (613.51 – 617.70) | 667.07 (665.01 – 669.14) | 8.4% |
| Other endocrine drugs | 199.61 (198.41 – 200.81) | 166.29 (165.25 – 167.32) | -16.7% |
|  | | | |
| **Obstetrics, gynaecology, and urinary-tract disorders medications** | **32,262.91 (32,250.38 – 32,275.45)** | **54,914.09 (54,901.44 – 54,926.74)** | **70.2%** |
|  | | | |
| Drugs for genito-urinary disorders | 12,062.99 (12,054.25 – 12,071.72) | 35,948.02 (35,935.82 – 35,960.22) | 198.0% |
| Treatment of vaginal and vulval conditions | 2,988.11 (2,983.55 – 2,992.68) | 4,297.58 (4,292.43 – 4,302.74) | 43.8% |
| Contraceptives | 17,210.83 (17,200.70 – 17,220.95) | 14,668.48 (14,659.48 – 14,677.47) | -14.8% |
| Drugs used in obstetrics | 0.99 (0.90 – 1.07) | 0.01 (0.00 – 0.02) | -98.6% |
|  | | | |
| **Malignant disease and immunosuppression medications** | **8,029.08 (8,021.80 – 8,036.37)** | **8,385.11 (8,378.07 – 8,392.16)** | **4.4%** |
|  | | | |
| Sex hormones and hormone antagonists in malignant disease | 3,995.71 (3,990.46 – 4,000.97) | 5,561.35 (5,555.52 – 5,567.18) | 39.2% |
| Drugs affecting the immune response | 1,921.79 (1,918.10 – 1,925.47) | 2,284.15 (2,280.35 – 2,287.95) | 18.9% |
| Cytotoxic drugs | 2,111.58 (2,107.73 – 2,115.44) | 539.61 (537.75 – 541.47) | -74.4% |
|  | | | |
| **Nutrition and blood-related products** | **42,954.03 (42,936.83 – 42,971.22)** | **108,515.77 (108,490.76 – 108,540.77)** | **152.6%** |
|  | | | |
| Compound vitamin/mineral formulations | 104.83 (103.96 – 105.70) | 494.08 (492.30 – 495.86) | 371.3% |
| Vitamins | 15,602.63 (15,592.90 – 15,612.36) | 55,804.78 (55,792.16 – 55,817.41) | 257.7% |
| Foods | 143.15 (142.14 – 144.17) | 399.94 (398.33 – 401.54) | 179.4% |
| Minerals | 1,481.28 (1,478.04 – 1,484.52) | 3,845.97 (3,841.08 – 3,850.86) | 159.6% |
| Anaemias and some other blood disorders | 14,714.70 (14,705.20 – 14,724.20) | 35,188.51 (35,176.37 – 35,200.65) | 139.1% |
| Health supplements | 74.66 (73.93 – 75.39) | 131.99 (131.07 – 132.91) | 76.8% |
| Oral nutrition | 7,969.04 (7,961.78 – 7,976.30) | 10,382.54 (10,374.79 – 10,390.30) | 30.3% |
| Fluids and electrolytes | 2,170.17 (2,166.26 – 2,174.08) | 2,142.66 (2,138.98 – 2,146.34) | -1.3% |
| Metabolic disorders | 11.52 (11.23 – 11.80) | 11.32 (11.05 – 11.59) | -1.7% |
| Other health supplements | 644.52 (642.38 – 646.67) | 113.95 (113.09 – 114.81) | -82.3% |
| Intravenous nutrition | 0.23 (0.19 – 0.27) | 0.02 (0.01 – 0.03) | -93.4% |
| Bitters and tonics | 37.29 (36.78 – 37.81) | 0.01 (0.00 – 0.02) | -100.0% |
|  | | | |
| **Musculoskeletal and joint diseases medications** | **59,759.91 (59,746.76 – 59,773.06)** | **56,369.73 (56,357.12 – 56,382.34)** | **-5.7%** |
|  | | | |
| Drugs used in neuromuscular disorders | 1,551.75 (1,548.43 – 1,555.06) | 2,639.20 (2,635.13 – 2,643.28) | 70.1% |
| Drugs for the relief of soft-tissue inflammation | 9,034.11 (9,026.42 – 9,041.80) | 13,522.55 (13,513.86 – 13,531.25) | 49.7% |
| Drugs used in rheumatic diseases and gout | 49,174.05 (49,160.64 – 49,187.46) | 40,207.97 (40,195.51 – 40,220.44) | -18.2% |
|  | | | |
| **Eye medications** | **31,691.22 (31,678.75 – 31,703.70)** | **33,348.64 (33,336.65 – 33,360.62)** | **5.2%** |
|  | | | |
| Treatment of glaucoma | 11,923.94 (11,915.25 – 11,932.63) | 16,915.83 (16,906.30 – 16,925.36) | 41.9% |
| Miscellaneous ophthalmic preparations | 8,326.86 (8,319.45 – 8,334.27) | 8,979.81 (8,972.54 – 8,987.08) | 7.8% |
| Corticosteroids and other anti-inflammatory preparations | 4,605.27 (4,599.65 – 4,610.89) | 4,274.83 (4,269.69 – 4,279.97) | -7.2% |
| Mydriatics and cycloplegics | 209.97 (208.75 – 211.20) | 161.29 (160.27 – 162.31) | -23.2% |
| Local anaesthetics | 3.57 (3.41 – 3.73) | 1.86 (1.75 – 1.97) | -47.8% |
| Anti-infective eye preparations | 6,621.19 (6,614.52 – 6,627.86) | 3,015.02 (3,010.67 – 3,019.37) | -54.5% |
| Contact lenses | 0.42 (0.37 – 0.48) | - | n/a |
|  | | | |
| **Ear, nose, and oropharynx medications** | **18,844.39 (18,833.90 – 18,854.88)** | **20,726.21 (20,715.90 – 20,736.51)** | **10.0%** |
|  | | | |
| Drugs acting on the nose | 10,530.49 (10,522.26 – 10,538.73) | 14,816.04 (14,807.01 – 14,825.07) | 40.7% |
| Drugs acting on the ear | 4,373.68 (4,368.20 – 4,379.17) | 3,358.44 (3,353.86 – 3,363.02) | -23.2% |
| Drugs acting on the oropharynx | 3,940.21 (3,934.99 – 3,945.43) | 2,551.73 (2,547.72 – 2,555.74) | -35.2% |
|  | | | |
| **Skin medications** | **69,938.78 (69,926.49 – 69,951.08)** | **57,095.46 (57,082.88 – 57,108.05)** | **-18.4%** |
|  | | | |
| Vehicles and emulsifying agents | 37.04 (36.52 – 37.55) | 146.62 (145.65 – 147.59) | 295.9% |
| Sunscreens and camouflagers | 407.76 (406.05 – 409.47) | 790.86 (788.61 – 793.12) | 94.0% |
| Preparations for eczema and psoriasis | 2,357.49 (2,353.43 – 2,361.56) | 2,716.49 (2,712.35 – 2,720.62) | 15.2% |
| Antiperspirants | 106.40 (105.53 – 107.28) | 114.48 (113.62 – 115.34) | 7.6% |
| Miscellaneous topical preparations | 12.12 (11.82 – 12.41) | 12.34 (12.06 – 12.62) | 1.8% |
| Shampoo and other preparations for scalp conditions | 2,910.73 (2,906.22 – 2,915.23) | 2,685.06 (2,680.95 – 2,689.17) | -7.8% |
| Topical corticosteroids | 24,246.27 (24,234.77 – 24,257.76) | 20,974.89 (20,964.54 – 20,985.24) | -13.5% |
| Acne and rosacea | 3,877.25 (3,872.07 – 3,882.42) | 3,286.83 (3,282.30 – 3,291.37) | -15.2% |
| Topical local anaesthetics and antipruritics | 653.66 (651.50 – 655.82) | 537.18 (535.32 – 539.04) | -17.8% |
| Emollient and barrier preparations | 22,902.56 (22,891.29 – 22,913.83) | 18,162.29 (18,152.49 – 18,172.09) | -20.7% |
| Anti-Infective skin preparations | 9,114.85 (9,107.13 – 9,122.57) | 6,603.37 (6,597.06 – 6,609.69) | -27.6% |
| Topical circulatory preparations | 287.66 (286.22 – 289.10) | 186.32 (185.23 – 187.42) | -35.2% |
| Preparations for warts and calluses | 740.93 (738.63 – 743.22) | 421.36 (419.71 – 423.01) | -43.1% |
| Skin cleansers and antiseptics | 2,100.04 (2,096.19 – 2,103.88) | 422.30 (420.65 – 423.95) | -79.9% |
| Wound management products | 184.04 (182.89 – 185.19) | 35.06 (34.58 – 35.53) | -80.9% |
|  | | | |
| **Immunological products and vaccines** | **27,124.69 (27,112.77 – 27,136.62)** | **22,835.83 (22,825.15 – 22,846.50)** | **-15.8%** |
|  | | | |
| Vaccines and antisera | 27,117.90 (27,105.98 – 27,129.82) | 22,835.66 (22,824.99 – 22,846.33) | -15.8% |
| Immunoglobulins | 6.79 (6.57 – 7.01) | 0.16 (0.13 – 0.20) | -97.6% |
| Diagnostic vaccines | - | - | n/a |
|  | | | |
| **Anaesthesia** | **1,652.72 (1,649.30 – 1,656.14)** | **3,091.59 (3,087.19 – 3,095.99)** | **87.1%** |
|  | | | |
| General anaesthesia | 109.82 (108.93 – 110.71) | 562.74 (560.84 – 564.64) | 412.4% |
| Local anaesthesia | 1,542.90 (1,539.59 – 1,546.20) | 2,528.85 (2,524.86 – 2,532.85) | 63.9% |

n/a: not applicable

**Figure 1S: Prescription rates of all medications in England and Wales between 2004 and 2019**
